# Pluralistic task shifting for a more timely cancer diagnosis. A grounded theory study from a primary care perspective

**DOI:** 10.1080/02813432.2021.2004751

**Published:** 2021-12-10

**Authors:** Hans Thulesius, Ulrika Sandén, Davorina Petek, Robert Hoffman, Tuomas Koskela, Bernardino Oliva-Fanlo, Ana Luísa Neves, Senada Hajdarevic, Lars Harrysson, Berit Skjodeborg Toftegaard, Peter Vedsted, Michael Harris, The Örenäs Research Group

**Affiliations:** a Department of Clinical Sciences Malmö, Family Medicine, Lund University, Lund, Sweden; b Research and Development Centre, Region Kronoberg, Växjö, Sweden; c Department of Medicine and Optometry, Linnaeus University, Kalmar, Sweden; d Department of Design Sciences, Faculty of Engineering, Lund University, Lund, Sweden; e Department of Family Medicine, Faculty of medicine, University of Ljubljana, Ljubljana, Slovenia; f Departments of Family Medicine & Medical Education, Sackler Medical School, Tel Aviv University, Tel Aviv, Israel; g Department of General Practice, School of Medicine, University of Tampere, Tampere, Finland; h Department of Primary Health Care, Palma, Spain; i Patient Safety Translational Research Centre, Institute of Global Health Innovation, Imperial College London, London, UK; j Department of Community Medicine, Health Information and Decision, Faculty of Medicine, University of Porto, Porto, Portugal; k Department of Nursing, Umeå University, Umeå, Sweden; l School of Social Work, Faculty of Social Sciences, Lund University, Lund, Sweden; m Department of Emergency Medicine, Horsens Hospital, Horsens, Denmark; n Department of Clinical Medicine, Research Unit for General Practice, The Research Centre for Cancer Diagnosis in Primary Care, Aarhus University, Aarhus, Denmark; o College of Medicine & Health, University of Exeter, Exeter, UK; p Institute of Primary Health Care (BIHAM), University of Bern, Bern, Switzerland

**Keywords:** Cancer diagnosis; primary care; qualitative data; grounded theory

## Abstract

**Objective:**

To explore how cancer could be diagnosed in a more timely way.

**Design:**

Grounded theory analysis of primary care physicians’ free text survey responses to: ‘How do you think the speed of diagnosis of cancer in primary care could be improved?’. Secondary analysis of primary care physician interviews, survey responses, literature.

**Setting:**

Primary care in 20 European Örenäs Research Group countries.

**Subjects:**

Primary care physicians: 1352 survey respondents (2013-2016), 20 Spanish and 7 Swedish interviewees (2015–2019).

**Main outcome measures:**

Conceptual explanation of how to improve timeliness of cancer diagnosis.

**Results:**

*Pluralistic task shifting* is a grounded theory of a composite strategy. It includes *task sharing* – among nurses, physicians, nurse assistants, secretaries, and patients – and *changing tasks* with cancer screening when appropriate or cancer fast-tracks to accelerate cancer case finding. A *pluralistic dialogue culture* of comprehensive collaboration and task redistribution is required for effective pluralistic task shifting. Pluralistic task shifting relies on *cognitive task shifting,* which includes learning more about slow analytic reasoning and fast automatic thinking initiated by pattern recognition; and *digital task shifting,* which by use of eHealth and telemedicine bridges time and place and improves power symmetry between patients, caregivers, and clinicians. *Financial task shifting* that involves cost tracking followed by reallocation of funds is necessary for the restructuring and retraining required for successful pluralistic task shifting. A timely diagnosis reduces expensive investigations and waiting times. Also, late-stage cancers are costlier to treat than early-stage cancers. Timing is central to cancer diagnosis: not too early to avoid overdiagnosis, and never too late.

**Conclusions:**

We present *pluralistic task shifting* as a conceptual summary of strategies needed to optimise the timeliness of cancer diagnosis.

**Key points**
- Cancer diagnosis is under-researched in primary care, especially theoretically. Thus, inspired by classic grounded theory, we analysed and conceptualised the field:
- *Pluralistic task shifting* is a conceptual explanation of how the timeliness of cancer diagnosis could be improved, with data derived mostly from primary care physicians.
- This includes *task sharing* and *changing tasks* including screening and cancer fast-tracks to accelerate cancer case finding, and requires *cognitive task shifting* emphasising learning, and *digital task shifting* involving the use of eHealth and telemedicine.
- *Financial task shifting* with cost tracking and reallocation of funds is eventually necessary for successful pluralistic task shifting to happen.

## Introduction

Diagnosing cancer is heterogeneous, in that it depends on disease type, age, gender, socioeconomic and geographical context, and type of healthcare system [1–3]. Some cancers, such as breast cancer, colorectal cancer and prostate cancer, may be detected by screening in an early asymptomatic phase of the disease [4]. However, the majority of cancers are discovered by case finding: symptoms and signs of the cancer are assessed through consultations with health care professionals [5] and most cancer patients are first seen by a primary care physician [1,2].

Work-up of a cancer diagnosis often requires several different assessment methods, which may or may not include a physical examination [5]. These are followed by technical procedures which include diagnostic imaging techniques and blood tests [2]. A histological examination of body tissue or cells ultimately confirms the cancer diagnosis, except for some late-stage cancers, often in the elderly, which may only be discovered by diagnostic imaging or at autopsy [6].

Many countries have introduced fast-track systems for detecting cancer that are effective in improving case-finding if the symptoms and signs of patients meet specific fast-track criteria [3]. Investigations for suspected cancer can in some countries be done by centralised or specialised diagnostic services that target many diagnoses simultaneously for those patients who do not meet fast-track criteria and where case finding fails [3,7,8].

The complexity of the cancer diagnostic pathway described above implies that there are many opportunities for error and delay. These many issues need to be resolved to optimise the work-up processes involved.

The purpose of this study was to analyse, from a primary care perspective, how the timeliness of cancer diagnosis could be enhanced. Since theories on care improvement are rare but encouraged [9], we present a grounded theory to provide conceptual hypotheses to explain how we may achieve a more timely diagnosis of cancer.

## Methods

### Data collection

We collected data mainly from surveys of European primary care physicians. Secondarily we collected data from interviews with primary care physicians, from scientific literature data and from news articles and internet media.

We performed an online survey study of primary care physicians in 25 Örenäs Research Group centres in 20 countries between November 2015 and December 2016 (Bulgaria, Croatia, Denmark, England, Finland, France, Germany, Greece, Israel, Italy, Netherlands, Norway, Poland, Portugal, Romania, Scotland, Slovenia, Spain, Sweden and Switzerland).

The methodology and the development of the Örenäs Research Group survey is described elsewhere [3,10]. The overall response rate for the survey was 24.8%, ranging from 7.1%–65.6% between participating countries.

We also used data from an online survey by the International Cancer Benchmarking Partnership to Danish and Swedish primary care physicians in 2013 (translated from Danish by BST and from Swedish by HT) [11].

Data from a focus group interview study with Spanish primary care physicians in 2015 were also used (translated from Castilian by BOF) [12].

We further collected data from interviews with Swedish primary care physician researchers 2015-2019 who had participated in a detailed audit of the diagnostic process from electronic health records of six hundred patients diagnosed with bowel, lung, and urinary tract cancer (translated from Swedish by HT) [13].

The Spanish focus group data were transcribed from audio recordings, while the Swedish interview data were recorded in field notes. Scientific and popular literature on cancer diagnosis, including articles and comments from online sources, was also studied in the theory generation process. The scientific literature analysed in this study is given in the reference list.

### Subjects

#### Primary care physicians

The Örenäs Research Group survey qualitative data were retrieved from free text comments written by 1352 respondents from 20 European countries; free text comments in the International Cancer Benchmarking Partnership survey were written by 237 Danish and 165 Swedish respondents [11]; transcribed qualitative data from 20 Spanish focus group participants [12]; and qualitative data in field notes from seven Swedish individual interviews with primary care physicians [13] were also used as data for the analysis.

### Data analysis

Our analysis was inspired by classic grounded theory, which is the world’s most cited behavioural research method with 137,065 Google Scholar citations for its seminal publication (29 June, 2021) [14].

We primarily analysed free text survey responses to: ‘How do you think the speed of diagnosis of cancer in primary care could be improved?’ Thus, a preformed question was the basis analysis, which in line with classic grounded theory where a starting point of the analysis should not be set in advance.

Secondary data analysis of survey data, focus group data and individual interview data was done by applying the same grounded theory procedures as in recent studies, following the classic grounded theory ‘all is data’ dictum [15–20]. Classic grounded theory uses a mostly inductive approach to generate hypotheses that explains how participants in a studied area resolve their main concern. Grounded theory aims to generate conceptual theories presenting explanatory hypotheses that transcend cultural, temporal and contextual boundaries. Relevant and modifiable grounded theory concepts that work to explain what is going on should be able to fit in diverse settings and go beyond disciplinary and geographical borders [14,21–27].

A classic grounded theory research conceptualises ‘what is going on’ in the field of study by constantly comparing data during an iterative research process which involves open coding, memoing, theoretical sampling (data collection based on emerging hypotheses from the ongoing analysis), selective coding (coding and recoding particular data based on central concepts from the ongoing analysis), sorting (sorting memos according to relationships between concepts in the theory), re-sorting and then writing up the sorted memos into a working paper and eventually a publication [14,21–27].

Once the core category that explained what was going on in the data was generated, which in this study first was ‘pluralistic retasking’, but later renamed ‘pluralistic task shifting’, the analysis was delimited to the core category and related categories, and selective coding was done. Memoing, with the core category guiding the analytic work, then continued.

Following grounded theory rules, most of the conceptual literature was analysed at the end of the study [14,21–27].

The group of authors is multidisciplinary, comprising researchers in nursing (SH), social work and design (US), economic history (LH), and physician researchers (HT, DP, RH, TK, B O-F, ALN, BST, PV, MH). Our study was inspired by the concept of pluralistic dialogue, with numerous e-mail rounds, several telephone discussions and some face-to-face meetings knitting the group of authors together in the analytic process over a period of five years. This brought a collective intelligence perspective to the emergence of the theory [28].

A classic grounded theory study generates hypotheses for new theory based on thorough systematic analyses of large amounts of data, both empirical and interpreted, quantitative as well as qualitative. The quality of a classic grounded theory may be tried against the principles of ‘fit’, ‘work’, ‘relevance’ and ‘modifiability’ set forth by Glaser and Strauss [14] and Glaser [21–27]. ‘Fit’ has to do with how closely concepts fit the incidents they are representing. Achieving fit requires rigorous adherence to the constant comparison process, where incidents are compared to each other and to emerging concepts. A ‘relevant’ study deals with the real concern of the participants and captures attention. The theory ‘works’ when it explains how the problem, or main concern of participants, is being resolved and when it accounts for most of the variation in participants’ behaviour in the substantive area. A ‘modifiable’ theory is one that is never complete but can always be further developed when new relevant data are compared to existing data. A classic grounded theory is never right or wrong, it just has more or less fit, relevance, workability and modifiability, and readers of this paper may assess its quality according to these principles.

Descriptive and narrative data from the survey part of this study have been reported elsewhere [3,10].

### Ethics approval

Neither the mail survey nor the interview data in this study required formal research ethics approval according to Swedish law, but the Regional Research Ethics Committee in Linköping gave a positive advisory statement regarding the International Cancer Benchmarking Partnership survey (Diary number 2011/495-31). Local study leads of the Örenäs Research Group were asked to either gain ethical approval or obtain a statement that formal ethical approval was not needed in their jurisdiction [3,10].

## Results

### Rethinking cancer diagnosis by pluralistic task shifting

In this study, based on conceptualised data from the written reflections and ideas of many primary care physicians, but also literature data, we propose that a compound strategy of ‘pluralistic task shifting’ is the core variable that can explain what could be done to improve the timeliness of cancer diagnosis from a primary care perspective.

Table 1 gives an overview of the most important concepts in the study including the core variable Pluralistic Task Shifting and its sub-core variables.

**Table 1.** Overview of the most important concepts in the theory of Pluralistic Task Shifting for a more timely cancer diagnosis.

RETHINKING CANCER DIAGNOSIS: Making cancer diagnosis more timely by PLURALISTIC TASK SHIFTING includes TASK SHARING and CHANGING TASKS which requires a CULTURE of PLURALISTIC DIALOGUE and COGNITIVE and DIGITAL TASK SHIFTING All the above need FINANCIAL TASK SHIFTING

Table 2 provides definitions of the concepts used in the study.

**Table 2.** Definitions of concepts used in the study of the theory of Pluralistic Task Shifting for a more timely cancer diagnosis.

‘Rethinking cancer diagnosis’: the ‘main concern’ of the survey participants, based on the question ‘How do you think the speed of diagnosis of cancer in primary care could be improved?’.
‘Pluralistic task shifting’: the ‘core variable’ that explains what is going on - it is a resolution of the main concern, giving an overall explanation to how the timeliness of diagnosis of cancer in primary care could be improved. ‘Task shifting’ is the ‘rational redistribution of tasks among health workforce teams’ mostly described in healthcare in low-income countries according to WHO 2008 [29]. It is an ‘in-vivo’ concept from health care. In our context, it explains how tasks such as cancer diagnosis could be improved. ‘In-vivo’ means that task shifting is an existing concept used within the substantive area of scrutiny. Task shifting has been ‘emergently fitted’ to the data, meaning that we have hypothesised that task shifting explains and covers what the respondents and literature data are suggesting on how to improve the timeliness of cancer diagnosis.
‘Task sharing’: a property of task shifting that emphasises collaboration, team working and training.
‘Vertical task shifting and task sharing’: a process that involves staff at *different* levels of training and competence, for example shifting from community health workers to nurses, or from nurses to physicians.
‘Horizontal task shifting and sharing’: a process that involves staff at *similar* levels of training and competence, for example shifting and sharing from a physician in one speciality to a physician in another one.
‘Changing tasks’: a property of pluralistic task shifting that explains standardised cancer diagnostic pathways and screening programmes.
‘Culture of pluralistic dialogue’: an evolving cooperative dialogue among professionals crossing boundaries of disciplines. It focuses on patients/clients and service delivery. It is a requirement for a successful task shifting and sharing to develop. 'Pluralistic dialogue' is an emergently fitted, or ‘borrowed’, grounded theory concept explaining professionals collaborating by deconstructing and resynthesising thinking, rethinking professional responsibility and reframing team responsibility by breaking stereotypical images [30].
‘Cognitive task shifting’: a property of pluralistic task shifting emphasising ‘thinking cancer’. It includes the ‘fast thinking’ used in intuitive diagnosis and the ‘slow thinking’ prompted by algorithms.
‘Digital task shifting’: a property of pluralistic task shifting that emphasises telemedicine and eHealth.
‘Financial task shifting’: a prerequisite for pluralistic task shifting; it includes reallocating funds (‘money to follow’) and cost tracking (‘follow the money’).

Pluralistic task shifting is a conceptual name for the overarching pattern of behaviour suggested by primary care physicians in many countries and by literature data as a composite strategy to shrink organisational gaps, reduce structural bottlenecks and thus improve how cancer may be diagnosed in a more timely manner. ‘Pluralistic’ implies that the diagnostic tasks are many, and ‘shifting’ tells us that we must change how we undertake cancer diagnosis to achieve the goal of diagnosing cancer at the right time.

### Task shifting, sharing, and changing

**Task shifting** emerged early in the analysis to explain the multitude of reflections made by the participants in the Örenäs Research Group survey data. Task shifting has been in use for some time in health care, making it an ‘in-vivo’ concept, and it was defined by the World Health Organisation (WHO) as a ‘rational redistribution of tasks among health workforce teams’ mostly described in healthcare in low-income countries [29]. Task shifting fits well with how many respondents in our survey data wanted cancer diagnosis to work. Changing the focus of the tasks of primary care physicians from dealing with complaints that could be taken care of by other health care professionals to instead work more with unpacking potential cancer symptoms was mentioned by many respondents as a meaningful task shifting prioritisation from physicians to nurses. This is called vertical task shifting in the literature [31].

‘We should involve the nurses in gathering the patients’ medical history’

Polish primary care physician

‘We need better training of district nurses who initially assess the patient, often by phone’

Swedish primary care physician

Task shifting from hospital physicians to primary care physicians is called horizontal task shifting in the literature [31] and it was mentioned by many respondents.

‘We (primary care physicians) should directly refer to investigations without involving specialists who can do follow up of the diagnosed disease, unless when there is real diagnostic uncertainty, instead of doing routine tasks that the general practitioner can handle.’

Danish primary care physician

Shifting the focus of cancer diagnosis tasks from secondary care to primary care is a task shift motivated by longer waiting lists for hospital specialist care, while primary care physicians can offer a more timely access.

‘In hospitals the diseases stay, and the people come and go; in general practice, the people stay and the diseases come and go.’ [32]. This expression illustrates that primary care physicians already know most of their patients’ background and this can promote timeliness of cancer diagnosis.

***Task sharing*** as collaboration.

Task sharing between different health care professionals in primary and secondary care requires improved communication, collaboration and true cooperation, with a need for a dialogue culture.

Task sharing with the public and the patients by information and teaching about cancer alarm signs and symptoms was also mentioned as a way of speeding up cancer diagnosis.

‘More time for patient education and prevention, so that patients report faster on their own with worrying symptoms’

Polish primary care physician

‘Better health education for the population about alarm signals.’

Portuguese primary care physician

‘Safety netting’ was mentioned to ensure the communication of test results, meaning that physicians and patients share responsibility of the task of monitoring incoming results from diagnostic tests such as imaging and laboratory tests [33] as well as changed symptoms and new bodily sensations.

‘Normally, a follow-up physician appointment is booked, but I am also asking the patient to phone, that is doubled safety.’

Danish primary care physician

Respondents often suggested that task sharing could be achieved by use of digital tools in the form of e-mails, chat functions and overarching electronic health records, to minimise thresholds between primary and secondary care. We call this 'digital task shifting', see below.

**Task changing** by standardised diagnosis pathways.

Task changing is seen in many countries (such as Denmark, Norway, Sweden and the UK) that have introduced cancer fast-track systems for diagnosing cancer that work well if the symptoms and signs of patients meet the fast-track criteria. Centralised diagnosis procedures or specialised diagnosis services that target many diagnoses simultaneously, called Rapid Diagnostic Centres in the United Kingdom [34] and diagnostic centres in Denmark [8] and Sweden, serve patients who do not meet the fast-track criteria.

Task changing in the form of screening asymptomatic people for bowel, breast, cervical and prostate cancer is also a standardised diagnosis pathway which already exists in many, but not all, jurisdictions in the 20 surveyed countries [4].

**Digital task shifting** is defined as information- and communication technology (ICT)-based task sharing and shifting. Triaging using digital tools is already done by telemedicine care providers and can improve timeliness of cancer diagnosis [35].

Telemedicine and eHealth solutions for targeting the right person to screen or to investigate, and by making better use of electronic health records, could eventually improve the cancer work-up efficiency.

“Information and Communication Technology support directly in the patient records [is needed] – today we do not have many support tools within the electronic health records”

Swedish primary care physician

Digital task shifting could be achieved by better use of e-mails, chat functions and overarching electronic health records to minimise barriers between primary and secondary care. There is a huge potential for increased care task collaboration efforts, if we make better use of the advantages of ICT and telemedicine to bridge time and place.

‘With the powerful and fast ICT of today we have the potential for ultrafast diagnosis, but we still rely on analogue slow technique’

Swedish primary care physician

**Pluralistic dialogue culture.** Task shifting, task changing and task sharing between and within professional groups and with patients requires an attitude of rethinking where dialogue is necessary. And since the tasks are many, the dialogue must be pluralistic (Table 2). So, creating a collaborative dialogue work culture, where primary care physicians and specialists would meet in real life or by digital tools, was mentioned by several physicians as a way of improving task shifting and sharing.

‘By creating an informal meeting culture between GPs and specialists, so they know each other personally.’

Dutch primary care physician

‘Allowing virtual consultations with ‘end specialists’ to validate malignancy diagnosis’

Israeli primary care physician

**Cognitive task shifting** involves rethinking attitudes to, and awareness of, diagnostic reasoning.

Caregivers and patients are learning more about cancer diagnosis and how cancer may be discovered in primary care, where the vast symptom flow is mostly of a benign nature.

Health care professionals may benefit from reflecting on how they perform diagnostic reasoning. According to the dual process theory of cognition, it is relevant to be aware of whether System 1 or System 2 is used [36]. ‘System 1’ diagnostic reasoning is based on fast intuitive thinking, induced by pattern recognition which involves ‘gut feelings’.

Gut feeling detection depends on a number of patient characteristics.

Either the patient signals immediately entering the room, or the patient comes with relatives, or the patient can signal by body language, facial expression, skin colour, or being a frequent attender or not.’

Summary memo from focus group with Spanish primary care physicians

'‘System 2’ diagnostic reasoning is analytic and involves slow rational thinking in algorithms, in this context using traditional cancer case-finding diagnosis. Cognitive task shifting seeks to increase clinicians’ awareness of these two systems of diagnostic reasoning. The ability to alternate between them is crucial for avoiding diagnostic delay. Hence a more timely diagnosis can be achieved:

‘By listening carefully to patients and thus recognising possible red flags or gut feelings.’

Dutch primary care physician

*Task shifting as time management*. Good cancer diagnosis involves optimal use of time. ‘Not too early’ to avoid over-diagnosis and ‘never too late’. Since time is a limited resource and cancer is often progressive and life threatening unless treated in time, reducing time intervals is what better diagnosis and treatment provides for cancer patients. So, task shifting cancer diagnosis should have optimal time management as a goal.

‘Time to listen to patients, better opportunity to have a quick consultation with a GP’

Danish primary care physician

**Financial task shifting** relies on reallocation of funds from hospital care to primary care. ‘Following the money’ and the need for ‘money to follow’ explain what underlies the necessary care restructuring to improve diagnosis timeliness. Task shifting thus involves health care reorganisation and accompanying budget rethinking or refinancing. ‘Following the money’ means tracking costs and thereby tracing structures and processes that need to change. By ‘following the money’ in the billing of medical procedures and tests, we have found evidence of short-sighted strategies in cancer diagnosis. These are not cost-efficient from a sustainable budget perspective.

‘Electronic Health Records…. focus too much on billing and solving how to bill most efficiently while solving the health issues become secondary.’

Primary care physician working in both US and Europe

As an example, primary care physicians in some countries were not reimbursed for some tests, for example prostate specific antigen, PSA. This lack of reimbursement delays cancer work-up, slows down the diagnosis process, and since cancer is more expensive to treat at a late stage than at early stages this costs more in the long term. So, by this economic logic, early cancer diagnosis is always better than late, except in relation to cancers where a 'watchful waiting' approach is used.

‘Money to follow’ indicates that refinancing, using financial incentives and billing for tests and procedures for cancer diagnosis, are necessary for the restructuring. This includes covering the costs of comprehensive training of those who will be able to have tasks shifted to them, for example nurses and healthcare assistants.

‘Increase funding for cancer diagnostic tests (tumour markers, colonoscopy, gastroscopy, radiographs) - currently, the funding is insufficient and as a result, PSA is rarely measured.’

Polish primary care physician

A few survey respondents from countries with little screening activity wanted compulsory cancer screening.

‘Gynaecology examination and mammographic screening should be made compulsory for all women regardless of their age’.

Bulgarian primary care physician

One primary care physician (from a context with no cancer screening available) wrote that if patients would not attend screening, they should get penalised by losing their health insurance. However, mandatory screening is a task shift that, according to several survey respondents and the literature, would risk overdiagnosis and overtreatment [37].

#### Task shifting nihilism

Overdiagnosis and overtreatment was mentioned by many respondents. Some were concerned that the changes necessary for earlier diagnosis could harm patients through over-treatment and unnecessary anxiety.

‘Not relevant [to diagnose cancer early]. Cancer diagnosis is a difficult balancing act between under- and overdiagnosis. Faster cancer diagnosis will also give more overdiagnosis.’

Danish primary care physician

But shifting the screening task from asymptomatic people to primary care patients that are ‘risk factor targeted’ might eventually reduce the risk of overdiagnosis and increase the cost benefit. Targeting people at risk could be done by using machine learning on electronic health record data or electronic surveys [38].

Some respondents were happy with the existing diagnostic speed and were more worried about overdiagnosis and the harm that is associated with finding cancers which may not need to be treated, or if treated would result in unnecessary suffering. This was especially true for respondents in countries, such as the United Kingdom, with fast-track diagnosis systems already in place.

#### Shifting diagnosis infrastructure

Faster access to tests (imaging, endoscopic and blood tests) was mentioned by many respondents as a way of speeding up the diagnosis of cancer. This could either be part of a task shift from secondary care to primary care physicians, or task sharing between them.

In some countries, primary care physicians had poor access to many of cancer work-up and diagnostic procedures. They needed to rely on secondary care specialists to get testing and imaging done, and this often resulted in long waits.

Ultrasound is an imaging option that few primary care physicians had access to. Improving access to ultrasound, either by easier referral or by primary care physicians doing ultrasounds themselves, was mentioned as a task that could speed up cancer diagnosis.

‘The choice of performing ultrasound scans by yourself or funded by the National Health Fund’.

Polish primary care physician

Bypassing secondary care specialists to get access to the diagnosis infrastructure was mentioned by many respondents, and this task bypassing is a shift that already happens in the fast-track systems in some countries.

Point-of-care testing was available in some countries but not for all tests, and in some countries with limited availability. More point-of-care testing would eventually speed up diagnosis according to many respondents, especially if cost issues could be addressed.

Task sharing between primary care physicians and secondary care specialists could be eased by ‘hotlines’ by telephone, e-chat, or e-mail to achieve smoother and faster communication between primary care physicians and specialists. This way of overcoming long waits and delays in diagnosis is an example of digital task shifting within a dialogue culture.

To achieve all these task shifts by sharing and changing tasks in the cancer work-up processes, many respondents emphasised the need to shift or redistribute the financing, and the physical and regulatory infrastructure of the health care system in general and of primary care in particular. Also, by reducing bureaucracy, corruption, and in some countries eliminating disincentives to refer patients or perform tests was mentioned to enable task changing and shifting to speed up cancer work-up routes.

This infrastructure shifting would help primary care to implement more point-of-care testing, facilitate the access to imaging and endoscopic procedures, and eventually improve the status of primary care.

## Discussion

In this grounded theory study on how to improve the speed of cancer diagnosis, an overall multivariate strategy of pluralistic task shifting emerged from the ideas of many primary care physician respondents across 20 countries and literature data. Pluralistic task shifting expands the concept of task shifting which was in forefront for the future of primary care according to the WHO:

‘…I see *task shifting* as the vanguard for the renaissance of primary health care…’

Margaret Chan, WHO Director General 2006–2017

Our prime data were written suggestions in a survey from the Örenäs Research Group. Additional data came from an International Cancer Benchmarking Partnership survey, Spanish and Swedish interviewees, and literature which included a WHO report on task shifting [29]. In many Anglo-Saxon and Nordic countries, as well as in the Netherlands and Slovenia, vertical task shifting from physicians to nurses in primary care has been in place for decades, with an emphasis on chronic disease management and prevention [39,40].

The respondents’ views in our study were conceptualised as pluralistic task shifting - suggesting that many things need to be done differently to achieve the goal of a more timely diagnosis for cancer patients. Task shifting and task sharing are key strategies that involve reorganising the health care workforce to provide the cancer services necessary to ease bottlenecks in the diagnostic process. Rethinking cancer diagnosis through pluralistic task shifting could be explained theoretically as a Basic Composite Strategy [21,23,26]. Functional dimensions of task shifting are digital task shifting by optimising digital tools, telemedicine and e-health, restructuring task shifting by default assessment procedures such as cancer fast-tracks and screening, and cognitive task shifting by training and fast and slow thinking in cancer case finding. Financial task shifting, with cost tracking (‘following the money’) and reallocating funds (‘money to follow’), are fundamental conditions for successful pluralistic task shifting.

That said, task shifting cancer diagnosis will only be achievable if someone is willing to pay the price. Thus, pluralistic task shifting not only requires an acceptance of organisational cultural change but also requires a comprehensive health economic perspective. It is necessary to develop financial incentives to achieve a more timely diagnostic process for cancer in primary care across many countries and jurisdictions. However, these incentives are intrinsic, in the sense that if we view costs across the whole health and general economic systems, it almost always costs less to manage a cancer that has been diagnosed earlier. Thus, more money in the health care system may not be required to achieve a more timely diagnosis of cancer [41,42].

To achieve pluralistic task shifting, a change in workplace culture involving pluralistic dialogue is suggested. Pluralistic dialogue is a concept discovered in a New Zealand grounded theory of hospital teamwork [30] and became part of our theory at an early stage as an ‘emergent fit’ (grounded theory jargon for ‘borrowing’ either earlier grounded theory concepts or *in vivo* concepts) [21,23]. Pluralistic dialogue explains how professionals succeed in collaborating by different strategies such as deconstructing and resynthesizing thinking, rethinking professional responsibility and reframing team responsibility. This eventually leads to the breaking of stereotypical images involving negotiating service provision.

In a Swedish grounded theory study of interactions between primary care physicians and patients in the context of standardised cancer pathways, ‘negotiating bodily sensations’ explained the reconciliation of the patients’ and the physicians’ expertise [43] and emphasises the tasks of patients and their unique role in diagnosing cancer.

Pluralistic task shifting shares some properties with the grounded theory of balancing cancer care [44], which explains problem-solving strategies of health care professionals in sensing patients’ symptom signals and gauging them against existing diagnostic and therapeutic resources. The balancing outcome is characterised by a compromise, at best an optimised situation, at worst a deceit.

An important condition for task shifting to happen is funding allocation or ‘money to follow’. Thus, one answer to the question ‘Why should we be task shifting cancer diagnosis?’ comes from the value-based care model [45], based on the assumption that ‘health systems should seek to obtain the maximum possible value for the health of people for every dollar they spend’ [46]. By cost tracking (‘following the money’), we can reveal costly bottlenecks and inefficient care processes. ‘From clinical pathways to care delivery value chains’, ‘promoting the right care and reducing medical overuse’ and eventually ‘turning a fragmented model into another integrated model’ are processes suggested by the value-based care model [47]. Similarly, pluralistic task shifting fits with the disruptive innovations concept from a design thinking perspective on health care innovations [48] explaining how existing structures become obsolete as a result of innovative improvements.

There are indeed problems with task shifting and we hypothesise this as especially caused by it being implemented outside of the context of a dialogue culture, as shown by Malterud, pointing to issues with patient safety when secondary care horizontally and one-sidedly shifts tasks to primary care [31].

Choosing experts, ‘elsewhereism’ of experts, and power symmetry issues were core concepts discovered in the seminal grounded theory ‘Experts vs Laymen’ [49]. Digital task shifting has improved the potential for contact between caregiver experts and layman patients, and between experts in primary and secondary care, by bridging time and place [50]. This reduces or modifies ‘elsewhereism’ and alters power symmetry, often to the advantage of the layman.

Cognitive task shifting as ‘thinking cancer’ in every primary care consultation was suggested by Holtedahl and includes ‘thinking cancer epidemiology’, ‘thinking organ-based symptoms’ and ‘avoiding diagnostic traps’ [51,52]. This belongs to the slow analytic ‘System 2’ diagnostic reasoning [36] which fits well with teamwork and thus pluralistic dialogue. It also aligns with the growing evidence for the use of cancer risk scores in primary care [1,53]. In Wales, ‘ThinkCancer!’ was an educational behaviour change aimed at the whole general practice team, designed to ensure timely diagnosis of cancer consisting of teaching and awareness sessions, the appointment of a ‘safety netting champion’ and the development of a bespoke ‘safety netting plan’ [54].

The conclusion of a Norwegian qualitative study of vertical task shifting in a haematology department fits well with our pluralistic task shifting theory:

‘Task shifting from doctors to nurses… requires not only development of technical skills but also complex changes in organisation, clinical routines and role identity. Educational and organisational interventions to build a team-oriented culture could potentially increase the possibility of successful task shifting and stimulate nurses to take on untraditional responsibilities. Environmental restructuring to support doctors using their time in activities only doctors can perform may be needed to realise potential efficiency gains’. [55]

## Strengths and limitations

This is, to our knowledge, the first grounded theory of cancer diagnosis from a systemic strategic perspective. Strengths include the rich qualitative data and large sample size sitting behind the explanatory concepts and the contextual scope of a grounded theory. Another strength is the collaborative learning process from a diverse group of expert analysts aspiring to achieve a collective intelligence outcome. The convergence of ideas from different research angles resulted in a conceptual theory that we hope can be understood and used across multiple disciplinary perspectives.

We only collected survey data from physicians, resulting in homogeneity of the survey population. However, the shared knowledge of the 1352 primary care physicians from 20 different health systems from countries spread geographically, and the analysis of multidisciplinary literature, yielded a coherent set of data, giving a primary care perspective that was not only international, but was also derived from heterogenous sources.

There are limits that come with a grounded theory which is not factual description but a set of conceptual hypotheses yet based on a large amount of data. Not everyone agrees with the importance of this type of conceptual theoretical knowledge in a ‘world run by description’ [56].

Another limitation of this study is that we mostly used physician survey data. Yet, the constant comparison procedures of grounded theory can compensate for particularistic bias. The different categories that emerged from attitude patterns in the survey data were repeatedly compared and carefully fitted with interview data and literature records on task shifting and sharing [57–60]. This leads us to conclude that the survey data were rich enough to allow conceptualisations that are relevant to other cultural and clinical settings.

## Meaning of the study

Pluralistic task shifting may be just an academic phrase or concept, but to be able to change structures and work processes in health care we need to change the language (and talk with each other) [61]. If we cannot formulate in abstractions what needs to be done, our arguments will be too descriptive and particularistic. By conceptualising we can better understand the world we live in and how to achieve the necessary change. ‘The role and value of theory in improvement work in healthcare has been seriously underrecognized’ [9]. This quote argues for the utility of the grounded theory of pluralistic task shifting and eventually trying to apply it outside of the field of cancer diagnosis.

How should then pluralistic task shifting be initiated? Inspired by Elinor Ostrom, we think that improving the timeliness of cancer diagnosis is a ‘polycentric task’ [62]. This means that many different actors must be involved in pluralistic task shifting that will only succeed through a ‘bottom-up’ process. Thus, it needs to be initiated by primary care organisations and their patients. Those who manage and use the care on a day-to-day basis can best see where there is the most need for change and amendments.

## Conclusions

Pluralistic task shifting is trying to answer the question ‘how may current cancer diagnosis be improved’ by conceptualising the thinking of many primary care physicians as well as literature data.

Pluralistic task shifting for more timely cancer diagnosis means that many things must be done differently, by a variety of actors, to discover and act on possible cancer at the right time, to the ultimate benefit of patients and citizens. We can achieve this demanding goal by optimising the use of technology, human resources and finances reflecting the task shifting dimensions digital, cognitive, and financial task shifting within a culture of pluralistic dialogue.

As the issues around cancer diagnosis are complex, unpacking the complexities informs our understanding of the problems. The challenge is to make this understanding help stakeholders to improve our health care systems for patients with cancer.
